# Comparison of Biostimulation versus Bioaugmentation with Bacterial Strain PM1 for Treatment of Groundwater Contaminated with Methyl *Tertiary* Butyl Ether (MTBE)

**DOI:** 10.1289/ehp.6939

**Published:** 2004-12-08

**Authors:** Amanda E. Smith, Krassimira Hristova, Isaac Wood, Doug M. Mackay, Ernie Lory, Dale Lorenzana, Kate M. Scow

**Affiliations:** ^1^Department of Land, Air and Water Resources, University of California, Davis, California, USA; ^2^Port Hueneme Naval Construction Battalion Center, Oxnard, California, USA

**Keywords:** bioaugmentation, biodegradation, bioremediation, groundwater, *in situ* remediation, microbial ecology, MTBE, pollutants

## Abstract

Widespread contamination of groundwater by methyl *tertiary* butyl ether (MTBE) has triggered the exploration of different technologies for *in situ* removal of the pollutant, including biostimulation of naturally occurring microbial communities or bioaugmentation with specific microbial strains known to biodegrade the oxygenate. After laboratory studies revealed that bacterial strain PM1 rapidly and completely biodegraded MTBE in groundwater sediments, the organism was tested in an *in situ* field study at Port Hueneme Naval Construction Battalion Center in Oxnard, California. Two pilot test plots (A and B) in groundwater located down-gradient from an MTBE source were intermittently sparged with pure oxygen. Plot B was also inoculated with strain PM1. MTBE concentrations up-gradient from plots A and B initially varied temporally from 1.5 to 6 mg MTBE/L. Six months after treatment began, MTBE concentrations in monitoring wells down-gradient from the injection bed decreased substantially in the shallow zone of the ground-water in plots A and B, thus even in the absence of the inoculated strain PM1. In the deeper zone, downstream MTBE concentrations also decreased in plot A and to a lesser extent in plot B. Difficulties in delivery of oxygen to the deeper zone of plot B, evidenced by low dissolved oxygen concentrations, were likely responsible for low rates of MTBE removal at that location. We measured the survival and movement of strain PM1 in groundwater samples using two methods for detection of DNA sequences specific to strain PM1: TaqMan quantitative polymerase chain reaction, and internal transcribed spacer region analysis. A naturally occurring bacterial strain with > 99% 16S rDNA sequence similarity to strain PM1 was detected in groundwater collected at various locations at Port Hueneme, including outside the plots where the organism was inoculated. Addition of oxygen to naturally occurring microbial populations was sufficient to stimulate MTBE removal at this site. In some cases, however, inoculation with an MTBE-degrading culture may be warranted.

The fuel additive methyl *tertiary* butyl ether (MTBE) has become a widespread environmental contaminant within the past 30 years. MTBE was the second most common volatile organic compound detected in wells monitored in urban areas nationwide between 1985 and 1995 (Clawges et al. 2000; Squillace et al. 1996). In California, for example, approximately 13,000 sites have hydrocarbon-contaminated groundwater, with more than 10,000 of these sites contaminated with MTBE (Happel et al. 1998). MTBE is very water soluble with a low sorption partition coefficient and thus is highly mobile in both groundwater and surface water. The pollutant is also moderately volatile, which can lead to redistribution and further contamination of the vadose zone, surface soils, and sediments.

There is little evidence that naturally occurring biodegradation processes, or intrinsic remediation, are substantial at many MTBE-contaminated sites, most of which are anoxic. Microcosm studies, however, have shown biodegradation to occur under some anaerobic conditions (Bradley et al. 2001a; Finneran and Lovley 2001). Aerobic biodegradation, on the other hand, is comparatively rapid if oxygen is present in or added to groundwater aquifers (Deeb et al. 2000b). Aerobic biodegradation of MTBE by native microorganisms has been measured in microcosm studies of MTBE- and oxygen-amended sediments (Bradley et al. 2001b) and groundwater samples (Salanitro et al. 2000; Wilson et al. 2002). Also, *in situ* MTBE bioremediation of a contaminated aquifer after addition of oxygen has been demonstrated at Port Hueneme Naval Construction Battalion Center (PH) in Oxnard, California, in both inoculated and uninoculated plots (Salanitro et al. 2000), and at Vandenberg Air Force Base, California, by native microbial communities (Wilson et al. 2002).

Our laboratory isolated an aerobic bacterium, strain PM1, that is capable of using MTBE as its sole carbon and energy source at relatively rapid rates. PM1 is a gram-negative rod and, based on 16S rDNA sequence similarity, is a member of the beta subgroup of Proteobacteria, in the family *Comamonadaceae*, and closely related to *Aquabacterium*, *Rubrivivax*, *Leptothrix*, *Ideonella*, and *Hydrogenophaga* (Bruns et al. 2001). PM1 rapidly mineralizes MTBE at concentrations up to 500 mg/L in laboratory cultures (Deeb et al. 2000a; Hanson et al. 1999) and degrades MTBE when inoculated into oxygenated groundwater or soil microcosms. Our goal was to evaluate whether *in situ* remediation using bioaugmentation with strain PM1 and oxygen addition was feasible to treat MTBE-contaminated groundwater.

Our specific objectives were to compare potential for and rates of MTBE removal from contaminated groundwater in field plots amended with oxygen. We compared bioaugmentation (inoculated with PM1) with biostimulation of naturally occurring MTBE-degrading microorganisms (not inoculated). We also measured the survival of strain PM1 in inoculated plots. The pilot study was conducted in an MTBE-contaminated aquifer at PH.

## Materials and Methods

### Biodegradation potential.

Cultures of strain PM1 were grown in mineral salts medium with MTBE as the sole carbon and energy source, as described previously (Hanson et al. 1999; Hristova et al. 2001). Cultures were incubated in 250-mL bottles sealed with Teflon-lined Mini-Nert (Dynatech Precision Sampling Corp., Baton Rouge, LA) caps at 25°C in the dark on an orbital shaker. After growth, cultures were centrifuged, and the cell pellets were washed twice, resuspended, and then used in inoculation. Inoculation densities were determined based on the standard optical density at 550 nm (OD_550_) versus cell plate count numbers. A microcosm study was conducted to determine the ability of strain PM1 to degrade MTBE in aquifer sediment samples from PH. Fifty-gram (dry wt) groundwater core samples were inoculated with 10^7^ strain PM1 cells per gram of sediment and 50 mL of groundwater. MTBE degradation was measured by analysis of 50-μL headspace samples using a Shimadzu GC-14A gas chromatograph equipped with a photonionization detector (Shimadzu, Columbia, MD). All experiments were performed in triplicate; concentrations of MTBE were calculated from a standard curve, and inoculated samples were compared with sterile controls containing 1% sodium azide (by mass).

### PH field trial.

**Figure 1:**
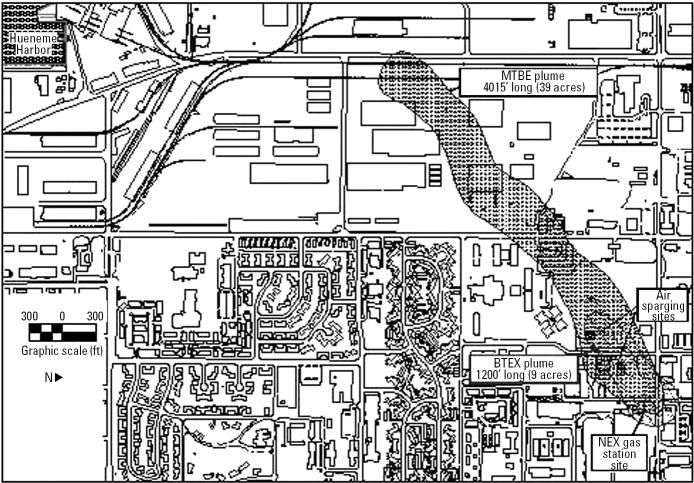
Map of MTBE plume at Port Hueneme Naval Construction Battalion Center in Oxnard, California. BTEX: benzene, toluene, ethylbenzene, xylene.

**Figure 2:**
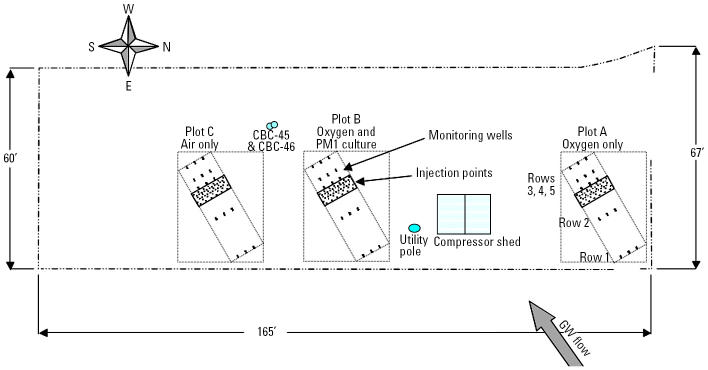
Layout of field test plots. Monitoring and oxygen injection well locations (both shallow and deep wells at many locations) are shown. GW, groundwater.

A field trial was initiated in a shallow, anoxic, MTBE-contaminated groundwater aquifer at PH (Figure 1). The geology and contamination at the site are described by Salanitro et al. (2000) from a bioaugmentation pilot study at PH using a different bacterial inoculant. The shallow aquifer consists of a clay-silt layer [0–3 m below ground surface (BGS)], fine- to medium-grain sand layer (3–6 m BGS), and a clay layer (beginning at 6 m BGS). The water table is approximately 2 m BGS, and groundwater flows southwest with a hydraulic conductivity of 53,000–120,000 L per day/m^2^. A 1,500-m plume resulted from a large release of gasoline containing MTBE from a gas station beginning in 1984. Test plots (each 2.7 m wide × 1.4 m long, separated from each other by 14 m) were installed 610 m down-gradient from the MTBE source. In two plots (A and B), groundwater was sparged with pure oxygen at two depths (between 3.7–4.3 and 5.2–5.8 m BGS) from 26 screened oxygen release wells. Oxygen was supplied using two 303-L air compressors with an Airsep Model AS80 oxygen generator (AirSep Corp., Rochester, NY). Approximately 12 m^3^ of oxygen was supplied to each plot per day. In a third plot, C, air rather than oxygen was added as an electron acceptor; these results are described elsewhere (Smith 2000). Each plot contained five rows of three monitoring well clusters, with each cluster consisting of two 1.9-cm-diameter wells: shallow (2.4–3.3 m) and deep (4.9–5.8 m). Wells were labeled by row number in each plot, depth (shallow or deep), and position in the row. For example, the deep well in plot B, located in the second row along the centerline, was called B22D (Figure 2). Two sets of wells were located up-gradient and three sets of wells down-gradient from the injection bed. Plot B was inoculated with strain PM1 as described below, whereas plots A and C were not. Groundwater was intermittently sampled for analysis of MTBE, oxygen, microbial populations, and other groundwater characteristics.

Oxygen delivery began late October 1999. Strain PM1 was added to plot B on 8 November 1999 (~ 10^9^ cells/mL in the injection solution of 220 gal of mineral salt media). Strain PM1 was injected using a Geoprobe unit (Geoprobe Systems, Salina, KS) at nine locations within the injection bed (shown as shaded area in each plot, Figure 2), in two rows interspaced between the oxygen-sparging wells.

Strain PM1 cultures for the field study were cultivated at the fermentation at Lawrence Livermore National Laboratory in Livermore, California. The cells were grown in a 1,500-L batch reactor on multiple inputs of 1,000 mg/L ethanol. After growth on ethanol, the cells were fed sequentially with 83, 192, and 255 mg/L MTBE. After removal of MTBE, the cells were harvested via continuous centrifugation and stored at 4°C until use. One week before injection, the cell paste was diluted in mineral salts media in four 55-gal plastic drums equipped with air sparging units and stirrers attached to a motor. Cells were fed 100 mg/L MTBE on a daily basis for a week and then transported to PH. At PH, the cells were mixed in a 1:1 ratio with groundwater from the site before injection.

### Sample collection and MTBE analysis.

MTBE samples were collected using Cole Parmer Masterflex peristaltic pumps (Cole-Parmer Instrument Co., Vernon Hills, IL). One well volume of groundwater, 450 mL for deep wells and 160 mL for shallow wells, was removed before sample collection. For MTBE analyses, groundwater samples were placed in 40 mL amber glass vials with septa tops containing 10% sodium azide as a sterilization agent. For DNA analyses, groundwater was collected with sterile tubing for each well, placed in 250-mL sterile plastic bottles, shipped on ice to the lab at the University of California, Davis, and frozen on receipt. MTBE was analyzed in 10-mL aliquots using purge-and-trap concentration followed by gas chromatography. Instrumentation included a Tekmar LSC 2000 Purge-and-trap, a VOCARB 3000 trap, Tekmar 2060 autosampler (Shimadzu), and a Shimadzu GC-14A gas chromatograph with a 15-m × 0.53-mm DB1 column (J&W Scientific, Folsom, CA) with a photonionization detector. This method provided a detection limit of 5 μg/L MTBE. A five-point calibration curve consisting of 0, 0.5, 1.0, 4.0, and either 2.0 or 10 mg MTBE/L was used for each run. The purge-and-trap procedure included a standby temperature of 35°C, purge time of 10 min, desorb preheat of 160°C, desorb of 4 min at 175°C, and bake time of 4 min at 260°C. The temperature program to reduce peak tailing included an initial temperature of 35°C for 5 min, temperature increase at a rate of 25°C/min, and final temperature of 150°C for 10 min.

### DNA extraction and analysis.

DNA was extracted from 5-mL microcosm or 130-mL groundwater samples using the same protocol. Bacterial cells were concentrated from water samples on white polycarbonate filters (diameter, 47 mm; pore size, 0.2 μm; type GTTP 2500, Millipore) (Fisher Scientific, Fair Lawn, NJ), placed on nitrocellulose support filters (47 mm, 0.45 μm) by applying a vacuum. After the tubes were frozen in liquid nitrogen, the filters were broken into small pieces, and DNA was extracted by bead beating and chloroform:isoamyl alchohol (24:1) as described by Hristova et al. (2001). The nucleic acids from the aqueous phase were concentrated and washed with 10 mM Tris-HCl, 1 mM EDTA (pH 7.8) in a microconcentrator (Centricon 100; Fisher Scientific), and the preparations were reduced to a final volume of 30 μL.

PM1-specific primers based on the internal transcribed spacer (ITS) region have been used to show presence of PM1 rDNA sequences in the groundwater. PM1-specific primers 1406F (5′-TGYACACACCGCCCGT-3′) and 1850R (5′-CGTAAGCCACTGACGCTT-3′) were designed based on alignments of sequences of the ITS and the flanking 16S and 23S rRNA genes of the ribosomal operon. The ITS PM1 primers amplify a 444-bp DNA fragment. Extracted DNA from pure cultures and groundwater samples was amplified using a Gene Amp 2400 thermal cycler (Applied Biosystems, Foster City, CA). The polymerase chain reaction (PCR) mixtures and conditions were as described by Hristova et al. (2003). PCR products were applied in 5- to 10-μL aliquots to 5% polyacrylamide gels (0.75 mm thick, 150 × 150 mm) and run on an electrophoresis unit at 150 V for 4 hr in 1 × Tris–borate/EDTA (pH 8.0) buffer. Gels were stained with SYBR Green and photographed through a yellow filter with a Kodak EDAS 290 CCD camera using Kodak 1D image analysis software (version 3.5.3; Scientific Imaging Systems, New Haven, CT).

We developed a quantitative real-time TaqMan PCR method for detection of 16S rDNA sequences specific to the MTBE-degrading strain PM1 (Hristova et al. 2001). The TaqMan method uses a fluorescent oligonucleotide probe with a 5′ reporter dye and 3′ quencher dye. During the PCR, the 5′–3′-nuclease activity of *Taq* DNA polymerase cleaves nucleotides from an oligonucleotide probe annealed to a target DNA strand. As the amplification reaction proceeds, more amplicons become available for probe binding, and consequently, the fluorescence signal intensity per cycle increases. The initial copy number is estimated from the exponential phase of product accumulation and by comparison with a standard curve. A 113-bp product was amplified using primers 963F and 1076R and probe 1030T (Hristova et al. 2001). Forty cycles of amplification, data acquisition, and data analysis were carried out using an ABI Prism 7700 Sequence Detector (PE Applied Biosystems). TaqMan PCR data were analyzed with Sequence Detector Software (version 1.7; Applied Biosystems). The threshold is defined as 10 times the standard deviation of the normalized fluorescent emission of the nontemplate control reaction. *C_T_* is the cycle at which a sample crosses the threshold, a PCR cycle where the fluorescence emission exceeds that of nontemplate controls. Three PCRs were performed for each environmental DNA extraction.

## Results

### Laboratory microcosms.

**Figure 3:**
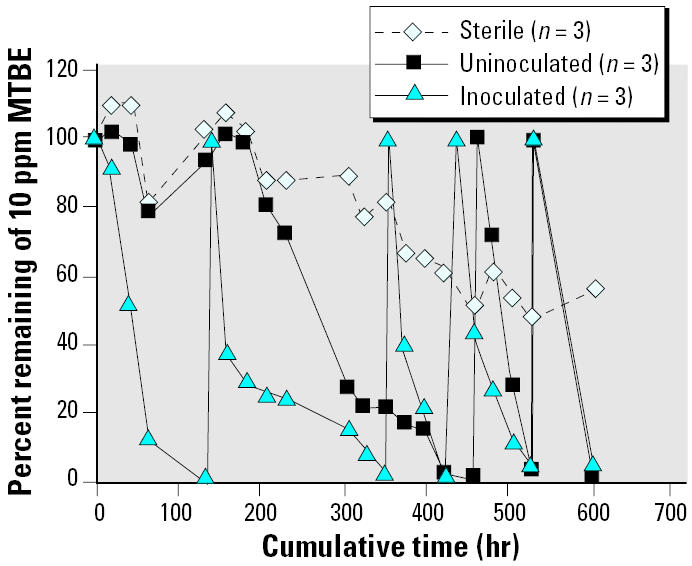
Effect of inoculation with strain PM1 on biodegradation of MTBE in PH groundwater: microcosm studies under aerobic conditions. The control microcosms contained 1% sodium azide as inhibitor. Each value is the average of three microcosms.

Addition of strain PM1 to groundwater sediments from PH spiked with 10 mg MTBE/L (10 ppm) resulted in more rapid MTBE removal than in uninoculated sediments (Figure 3). After 5 days, when the initial input of MTBE was degraded in the inoculated flasks, additional MTBE was added to the microcosms at the same concentration. MTBE degraded more rapidly than the first input of MTBE, suggesting an increase in the initial population density of MTBE-degrading organisms. The uninoculated samples also degraded MTBE, although after a longer lag period (200 hr, with complete degradation after 460 hr). Subsequent additions of MTBE in the uninoculated sediments, however, were degraded at rates comparable with those observed in inoculated flasks.

### MTBE removal in pilot study at PH.

Oxygen sparging in plots A and B was initiated in late October 1999; intensive sampling of dissolved oxygen in groundwater was conducted to determine when sufficient oxygen was present to support the activity of PM1. Modifications had to be made to the original design of the wells to increase oxygen delivery to locations where much of the MTBE was present. By early November, high concentrations of dissolved oxygen were measurable in almost all the shallow wells and in some, but not all, of the deeper wells at the site. Strain PM1 was added to plot B on 8 November 1999. We conducted frequent sampling of the center row wells (approximately every month) for oxygen and MTBE and less frequent sampling for microbiological analyses. To simplify the graphs, not all sampling dates are included; however, the other data show similar trends. The time of the injection of strain PM1 is referred to as time zero in the presentation of results.

**Figure 4:**
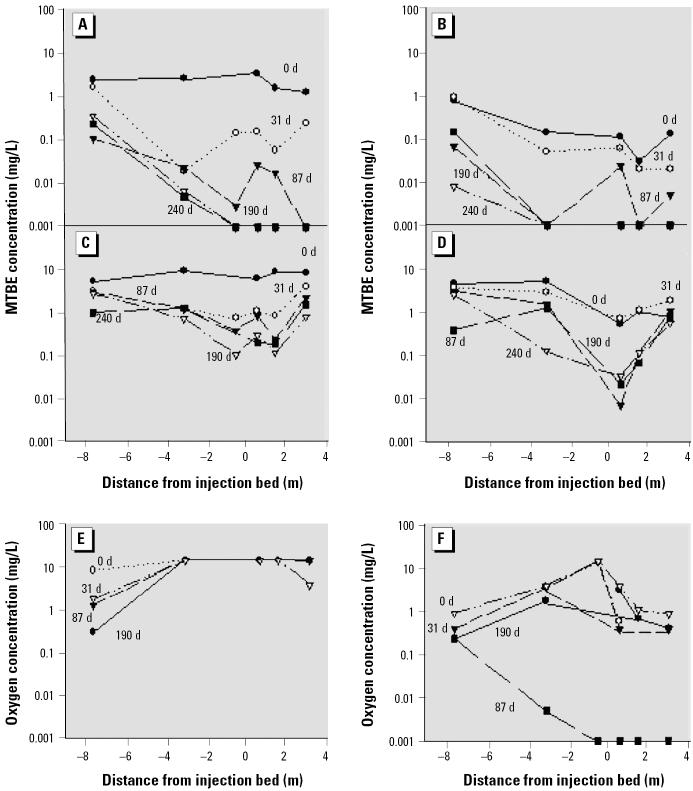
MTBE and oxygen concentrations in wells at plot B (inoculated) and plot A (uninoculated). Numbers on *x*-axis to the left of 0 are up-gradient; those to the right of 0 are down-gradient. (*A*–*F*) MTBE concentrations in plot B shallow wells (*A*) and deep wells (*C*); corresponding oxygen concentrations in shallow wells (*E* ) and deep wells (*F* ); and MTBE concentrations in plot A shallow wells (*B*) and deep wells (*D*). d, days.

Figure 4A and B shows MTBE removal along the direction of groundwater flow in the shallow centerline wells of plots B and A, respectively. The initial MTBE concentrations in the shallow zone ranged from 2.5 to 3.5 mg/L in plot B and were much lower in plot A (< 0.14 mg/L 2 m down-gradient of the oxygen release zone). Down-gradient of the oxygen release wells, MTBE concentrations decreased substantially in the shallow zone of both plots, even in the absence of strain PM1 (plot A). After 6 months of treatment, MTBE concentrations declined to 0.008 mg/L or nondetectable levels (< 0.005 mg/L) in plot A and to 0.09 mg/L or nondetectable levels in plot B.

In the deeper zone, initial MTBE concentrations ranged from 5 mg/L up-gradient to < 1 mg/L down-gradient in plot A and ranged from 5.7 to 9.3 mg/L in plot B. The differences in concentrations between plots A and B are likely due partly to spatial variability in MTBE concentrations but may also indicate that some treatment had already begun 1 month after oxygen injection began. Down-gradient MTBE concentrations decreased substantially in plot A to < 0.11 mg/L (Figure 4D) but only slightly in plot B (Figure 4C). Difficulties in delivery of oxygen to the deep zone in plot B, as evidenced by the low dissolved oxygen concentrations present (shown in Figure 4F), were likely responsible for low rates of MTBE removal at that location (Figure 4C). In addition, well pump tests indicated that groundwater flow was substantially slower in the shallow zones than in the deep zones and slower in plot B than in plot A (Smith 2000).

### PM1 detection and quantification in PH groundwater.

**Figure 5:**
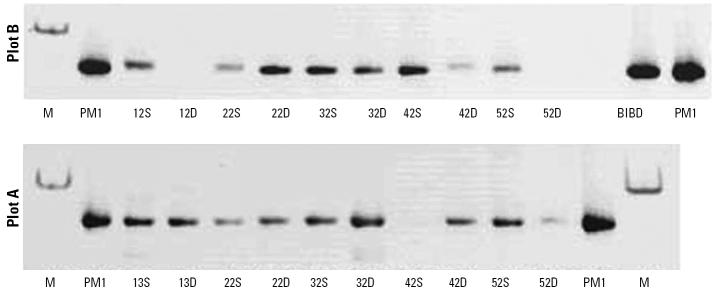
ITS analysis: PM1 presence in plot B (24 days after injection of strain PM1) and plot A (oxygen only). Abbreviations: D, deep well; S, shallow well. Inverted images of SYBR Green–stained polyacrylamide gels show PM1 ITS PCR products. Lane M, molecular marker; lane PM1, PM1 pure culture. Other lanes are labeled according to the well from which the DNA was extracted.

Three sets of PM1-specific primers (16S and ITS rDNA) were used to detect PM1 in groundwater samples. Using ITS-specific primers of DNA extracted from samples collected 24 days after injection of PM1, we detected PCR products with the expected length of 444 bp in all samples from plots B and A, with three exceptions (wells B-12-D, B-52-D, and A-42-S; Figure 5).

**Figure 6:**
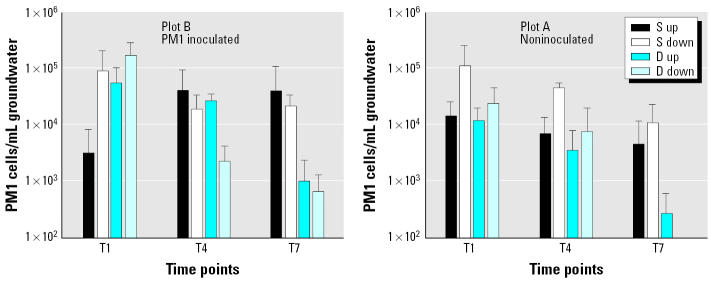
PM1 cell densities in PH groundwater from plot B and plot A as quantified by real-time PCR at three different time points: T1, 24 days after PM1 injection into plot B; T4, 120 days; T7, 240 days. Abbreviations: D, deep wells; S, shallow wells. Error bars indicate SD.

Using TaqMan PCR, we quantified the density of PM1 cells in samples (shallow and deep) from plots B and A (Figure 6A and B) at 24 days after injection (T1) of PM1. PM1 was detectable at densities ranging from 10^2^ to 10^5^/mL groundwater. Higher densities of PM1 were detected in deep wells, compared with shallow wells, in both test plots 31 days after inoculation. PM1 cell densities were quantified in some of the plot B wells that were up to one log order higher than those in plot A; however, this trend was not evident in other wells that showed similar densities of PM1. Cell densities declined substantially over the course of the field trial (120 days, 240 days; Figure 6) in both the shallow and deep zones. The specificity of the TaqMan PCR primers for strain PM1 was confirmed by sequencing the PCR products. Sequence analyses of 16S rDNA TaqMan PCR products obtained with extracted DNA from plots B and A showed 99–100% similarity with the bacterial strain PM1. There was a strong correspondence between positive ITS PCR detection of strain PM1 and TaqMan detection of PM1 in samples containing more than 10^3^ cells/mL. At lower densities, the ITS conventional PCR method was not sensitive enough to detect strain PM1.

A third set of PM1-specific primers were designed to target a different region of 16S rRNA molecule than that targeted by the TaqMan primers (Hristova et al. 2003). Using this set of 16S primers with denaturing gradient gel electrophoresis (DGGE) analysis provided additional evidence of the presence of PM1 in both plots A and B. DNA sequences from the 375-bp band (from plot A and plot B) were 99% similar to the sequence of PM1’s 16S rDNA (data not shown).

In summary, our results suggest the presence of a naturally occurring strain PM1 at PH. We could not determine, however, whether the presence of PM1 in plot A was due to movement from the inoculated plot (by some unknown mechanism, perhaps related to sparging) or if the organism was native to the site. We also tested whether PM1 was present in locations far removed from the field site. Eight groundwater samples were collected outside of the field-tested plots 240 days after injection of strain PM1: two of the samples close to our plots, three up-gradient, and three down-gradient of the plots (Table 1). DNA was extracted and tested for PM1 presence by ITS and TaqMan PCR. PM1 was detected by both PCR techniques in only one of the samples located far away (down-gradient) from the test plots (CBC-51). Two more samples, one close to plot B (CBC-60-CS) and one farther down-gradient (PHA4), showed PM1 presence by TaqMan real-time PCR.

We isolated a bacterial strain from PH site PHA4 (623 m down-gradient the test plots), where 5.7 × 10^3^ cells/mL PM1 is present in the groundwater as estimated by TaqMan PCR (Table 1). Sequencing of portions of the genome of this bacterial strain indicated that the new isolate was 100% similar to PM1 in the 16S rDNA and 99% in the ITS region. The isolate was able to degrade MTBE in laboratory microcosms but at rates far slower than those measured for strain PM1 (data not shown).

## Discussion

A major objective of our study was to determine if bioaugmentation with strain PM1 was necessary to support *in situ* MTBE removal from contaminated groundwater. Our results indicate that rates of MTBE removal were similar in both inoculated and uninoculated plots amended with oxygen. It is possible that strain PM1 and/or a naturally occurring PM1-like organism may be responsible for some of the biological removal of MTBE in the plots, as evidenced by detection of strain PM1 sequences in groundwater samples from both plots A and B. Results of controlled microcosm studies provide further evidence that oxygen additions to uninoculated PH aquifer sediments stimulate native MTBE-degrading organisms already present in these samples. After exposure of the native community to MTBE in microcosms, additional inputs of MTBE were degraded at rates similar to those measured for the pure culture of strain PM1.

In a field study of MTBE bioremediation also conducted at PH, Salanitro et al. (2000) compared rates of MTBE removal in two oxygen-sparged test plots, one that was inoculated with a mixed culture, MC-100 and the other not inoculated. Native MTBE-degrading organisms in the uninoculated plots were also capable of MTBE removal, similar to what we observed in our study, when provided with oxygen. A long lag period elapsed, however, before MTBE removal was detectable in the uninoculated plots; no such lag was observed in the bioaugmentation plot in the study by Salanitro et al. (2000). The absence of a lag in the uninoculated plots in our study may be due to a longer period of exposure, and possible acclimation, of communities to MTBE in our plots than in the plots in the earlier study by Salanitro et al. (2000).

An unexpected discovery of our study was the detection, in groundwater microbial communities collected outside of plot B, of DNA sequences identical (in two regions of the 16S rDNA and in the ITS region) to sequences of our MTBE-degrading bacterium, strain PM1. These PM1-like sequences were found in locations far removed from where strain PM1 was inoculated and in samples collected with sterilized equipment that had not been used for sampling of inoculated plots. This result suggested that an organism similar to strain PM1 occurs naturally in groundwater at PH. We also found PM1-like sequences in groundwater samples from an MTBE-contaminated aquifer at Vandenberg Air Force Base, located approximately 90 miles north of PH (Hristova et al. 2003), and demonstrated there that PM1-like population densities reflect MTBE concentration and the presence of oxygen. Kane et al. (2001) also detected PM1-like sequences in groundwater samples from two other MTBE-contaminated aquifers in northern California. Future effort will be directed at determining how widespread is the native PM1 organism in the environment and on linking MTBE biodegradation rates to the presence of native PM1.

A limitation of our study was the inability to distinguish between the laboratory strain of PM1 inoculated into plot B and the naturally occurring PM1-like organism, making it impossible to monitor the survival and movement of the introduced strain of PM1. Other regions of strain PM1’s genome will be targeted to try to identify new sequences that may discriminate the laboratory from naturally occurring organisms sharing the same 16S rDNA sequence.

Scale-up and practical application of treatment technologies for biostimulation of native microbial communities are promising and have been demonstrated at some locations. Bruce et al. (2002) reported success with a full-scale MTBE treatment system at PH, in both inoculated and uninoculated treatment beds, with oxygen delivery via sparging. Also, other methods of oxygen delivery have been successfully field tested and shown to provide oxygen while supporting *in situ* biotreatment of MTBE (e.g., Landmeyer et al. 2001; Wilson et al. 2002). Bioaugmentation with MTBE-degrading organisms may be appropriate for sites where there appears to be limited MTBE biodegradation potential or where accelerated MTBE removal is desired.

## Figures and Tables

**Table 1:** PM1 presence in PH groundwater wells as quantified by TaqMan real-time PCR.

	Groundwater well									
	CBC-1	CBC-10	CBC-42	CBC-61-CS	CBC-60-CD	B32S	B32D	CBC-51	PHB4	PHA4
Distance from plot B (m)	800	649	303	40	40	0	0	474	623	737
PM1 density (cells/mL)	< DL	< DL	< DL	< DL	2.2 ± 0.4 × 10^4^	4.6 ± 0.4 × 10^2^	3.0 ± 0.2 × 10^4^	1.9 ± 0.2 × 10^5^	5.7 ± 0.3 × 10^3^	< DL

< DL, below detection limit.
